# MetaGaAP: A Novel Pipeline to Estimate Community Composition and Abundance from Non-Model Sequence Data

**DOI:** 10.3390/biology6010014

**Published:** 2017-02-17

**Authors:** Christopher Noune, Caroline Hauxwell

**Affiliations:** School of Earth, Environmental and Biological Sciences, Queensland University of Technology, Brisbane City QLD 4000, Australia; chris.noune@connect.qut.edu.au

**Keywords:** bioinformatics, baculoviruses, metapopulation, meta-barcoding, MetaGaAP, HaSNPV-AC53, community analysis

## Abstract

Next generation sequencing and bioinformatic approaches are increasingly used to quantify microorganisms within populations by analysis of ‘meta-barcode’ data. This approach relies on comparison of amplicon sequences of ‘barcode’ regions from a population with public-domain databases of reference sequences. However, for many organisms relevant ‘barcode’ regions may not have been identified and large databases of reference sequences may not be available. A workflow and software pipeline, ‘MetaGaAP,’ was developed to identify and quantify genotypes through four steps: shotgun sequencing and identification of polymorphisms in a metapopulation to identify custom ‘barcode’ regions of less than 30 polymorphisms within the span of a single ‘read’, amplification and sequencing of the ‘barcode’, generation of a custom database of polymorphisms, and quantitation of the relative abundance of genotypes. The pipeline and workflow were validated in a ‘wild type’ *Alphabaculovirus* isolate, *Helicoverpa armigera* single nucleopolyhedrovirus (HaSNPV-AC53) and a tissue-culture derived strain (HaSNPV-AC53-T2). The approach was validated by comparison of polymorphisms in amplicons and shotgun data, and by comparison of predicted dominant and co-dominant genotypes with Sanger sequences. The computational power required to generate and search the database effectively limits the number of polymorphisms that can be included in a barcode to 30 or less. The approach can be used in quantitative analysis of the ecology and pathology of non-model organisms.

## 1. Introduction

Culture-independent molecular techniques to identify and quantify components of microbial communities have been facilitated by the use of next generation sequencing (NGS) [1,2].

Shotgun sequencing and whole or partial genome assembly uses algorithms comparing sequence data to public sequence databases (such as Genbank) [2,3,4,5,6]. ‘Barcode’ analysis uses PCR amplification of well-characterized regions (e.g., the 16S rRNA sub-unit in bacteria, internal transcribed space (ITS) of fungi or cytochrome oxidase) and comparison to sequence databases specific to those regions to determine taxonomic assignment and relative abundance of taxa in the community [2,7,8,9,10,11].

Both approaches are limited by available sequencing technology that relies on partial genome ‘reads’, and by the scope and accuracy of sequences in the reference databases. Shotgun sequencing and partial genome assembly is biased towards identification of dominant genotypes or taxa as a result of the limited read depth across multiple whole genomes [10,12,13]. Amplicon sequencing introduces bias resulting from gene copy number, selection of primers, and classification based on limited span of the genome [2,7,12,14]. Both depend on reference databases which contain sequences from the small proportion of organisms that have been sequenced and variable standards of validation. Furthermore, non-model organisms, for which sequence databases are not available or for which marker regions have not been identified, require custom solutions. This is a particular issue in analysis of viral metapopulations and quasispecies [15].

Baculoviruses (*Baculoviridae*) are invertebrate-specific double-stranded DNA viruses with a genome of between 80 kb to 180kb [16]. The nucleopolyhedroviruses (*Alphabaculoviruses*) are known to contain high levels of genotypic and phenotypic diversity within a single isolate [17,18,19,20,21].

Previous studies on within-isolate diversity used techniques such as in vitro and in vivo isolation of sub-populations to identify strains [19,22,23,24,25], but such culture-dependent approaches themselves select a sub-set of strains that are adapted to the selection method, such as growth in tissue culture [19,26,27]. Molecular approaches include restriction fragment length polymorphism (RFLP) in combination with quantitative polymerase chain reaction (qPCR) [28,29,30,31], and denaturing gradient gel electrophoresis (DGGE) [32,33,34]. DGGE cannot be used reliably to quantify relative abundance and both qPCR and DGGE rely on primers that may not detect all variants [14,33,34,35,36,37].

Shotgun sequencing can be used to assemble a consensus sequence for an isolate containing multiple strains, and the same data can then be used to identify polymorphisms across the genome to determine the relative abundance of a single polymorphism [38,39,40]. Shotgun data can also be used to infer an approximate total number of strains within an isolate and the relative abundance of taxonomic clusters of strains within this population [13,19], but cannot determine the relative abundance of individual strains or abundance of strains that may contain multiple polymorphisms distributed across fragmented reads.

In this paper, we describe the application and validation of stepwise sequencing and a metabarcoding software pipeline to identify and quantify within-isolate strain variants within a baculovirus model.

## 2. Materials and Methods

### 2.1. Viruses

The baculovirus isolate HaSNPV-AC53 was obtained from AgBiTech Pty Ltd., passaged once in *H. armigera* larvae and DNA extracted as previously described [17,39].

The strain variant HaSNPV-AC53-T2 was derived from the AC53 wild type by passage in tissue culture and DNA extracted as previously described [19,41].

### 2.2. Identification of High Density Polymorphic Regions in Shotgun Data

DNA extraction from the HaSNPV-AC53 wild-type isolate, shotgun sequence generation using the Ion Torrent PGM, and assembly of a consensus sequence (Genbank accession: KJ909666) were completed as previously described [42]. The Genome Analysis Toolkit v3.5 (GATK) (Broad Institute, Cambridge, MA, USA) ‘best practices’ pipeline was used to identify substitutions, insertions and deletions (polymorphisms) in the shotgun data which were filtered to exclude those with a minimum genotype quality of below 60 (0.0001% error) and minimum read depth of 20x coverage [38]. Polymorphisms were annotated using Geneious R9.1.5 (Biomatters, Auckland, New Zealand) and snpEff 4.2 [43,44].

### 2.3. Amplicon Sequencing and Validation of Sequence Polymorphisms

Primers were designed to amplify custom ‘barcode’ regions of 325 and 365 bp (i.e., less than the span of a single Ion Torrent PGM read) within each of two ORFs with different polymorphism density (Table 1): Baculovirus Repeated ORF-A (BRO-A) and DNA polymerase.

Both BRO-A and DNA Polymerase ‘barcode’ regions of the AC53 wild-type isolate and the BRO-A region of the HaSNPV-AC53-T2 strain were amplified from DNA using the Platinum Taq High Fidelity Super Mix kit (Life Technologies, Thermo-Fisher, Waltham, MA, USA) and an Eppendorf Pro S thermocycler (Eppendorf, Hamburg, Germany) as per the Platinum Taq standard method (Life Technologies, Thermo-Fisher, Waltham, MA, USA). NGS amplicon preparation and clean-up was completed as per the Life Technologies (Thermo-Fisher, Waltham, MA, USA) Ion Torrent PGM fusion primer manual. Shotgun sequencing was completed using an Ion Torrent PGM with a 318v2 chip and 400 bp chemistry.

Read quality was determined using FastQC 0.11.4 (Babraham Institute, Cambridge, UK) and any reads containing artefacts and/or quality less than Q20 were removed. Reads were trimmed to the expected amplicon size (Table 1) to remove primer regions using Fastx-toolkit 0.0.14 (Hannon Laboratory, Cold Spring Harbor, New York, NY, USA) [45,46]. Polymorphisms within the amplicon reads data were identified as described for the shotgun data and validated by comparison using vcf-compare within the VCFtools package (version 0.1.14) [47].

### 2.4. Sanger Sequencing

Both ‘barcode’ regions of the AC53 isolate and the BRO-A region of the HaSNPV-AC53-T2 strain were amplified using the forward primer in Table 1, the Mango Taq kit (Bioline, Meridian Bioscience, Cincinnati, OH, USA) and an Eppendorf Pro S thermocycler (Eppendorf, Hamburg, Germany). PCR products were then cleaned using an Isolate II PCR clean-up kit (Bioline, Alexandria, Australia) and labelled using a Big Dye Terminator (BDT) v3.1 kit (Applied Biosystems, Thermo-Fisher, Waltham, MA, USA). Labelled products were then precipitated using EDTA/ethanol as per the BDT v3.1 kit insert. Products were then sequenced using an ABI 3500 Genetic Analyzer (Applied Biosystems, Thermo-Fisher, Waltham, MA, USA).

### 2.5. Genotyping and Abundance Pipeline

Amplicon reads were mapped to the relevant consensus sequence for the 2 ORFs in the HaSNPV-AC53 genome and the BRO-A sequence for the HaSNPV-AC53-T2 strain using the BWA mem 0.7.12 algorithm with default settings to produce unsorted SAM files [48]. These unsorted SAM files were converted to sorted BAM files using SAMtools 1.3 [49]. The BAM headers were then corrected and the reference sequences and BAM files were indexed and a sequence dictionary was produced using samtools 1.3 and picard-tools 2.5.0 (Broad Institute, Cambridge, Massachusetts, USA) [50].

**Figure 1:**
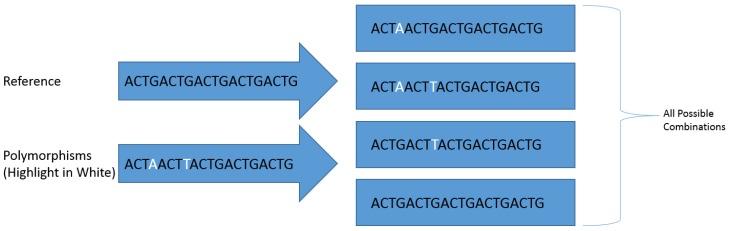
A visual representation of how the Biostars 175929 tool produces sequences containing all polymorphism combinations.

Polymorphisms were identified within the genome using the GATK HaplotypeCaller to produce a ‘genomic variant call format’ (gVCF) file with the following parameters: maximum read depth per site of 300,000 reads, 100 maximum alternate alleles per site, genotyping mode set to ‘discovery’,down-sampling set to ‘none’ and emit reference confidence set to ‘GVCF’. These files were then sorted to genotype and converted to a standard ‘variant call format’ (VCF) file using the GATK GenotypeGVCFs tool and hard-filtered using the GATK VariantFiltration tool to include only polymorphisms with a genotype quality (GQ) score greater than 60 (minimum 0.0001% error on a Phred scale). The final VCF file containing all the filtered polymorphisms within each ORF and the consensus sequence of each ORF were imported respectively into the Biostars 175929 tool as part of the JVarkit package [51] to produce a compressed fasta file database for each amplicon containing generated references sequences with every possible combination of identified polymorphisms (Figure 1). All generated reference sequences were renamed using the BBmap Renamer tool [52] to include the ORF from which they were derived and a numerical identification number.

The generated reference sequences were then indexed and used as the references to map amplicon reads using the BWA mem 0.7.12 algorithm with default settings to produce a SAM file [48]. The SAM files were then imported into Tablet 1.15.09.01 for visual and statistical comparison of the mapped amplicon sequence reads and the generated reference sequences [53,54]. The mapping statistics were produced using samtools 1.3 and sequences that contained less than 20x coverage (equivalent to a 1% error on a phred scale) or sequences with imperfect mapping (containing gaps) were excluded using kentUtils (version 302) and custom R scripts built with Microsoft R Open 3.3.1 [55,56,57]. Relative abundances of each identified genotype were calculated using Microsoft R Open 3.3.1 (Microsoft, Redmond, WA, USA) [55,57].

**Figure 2:**
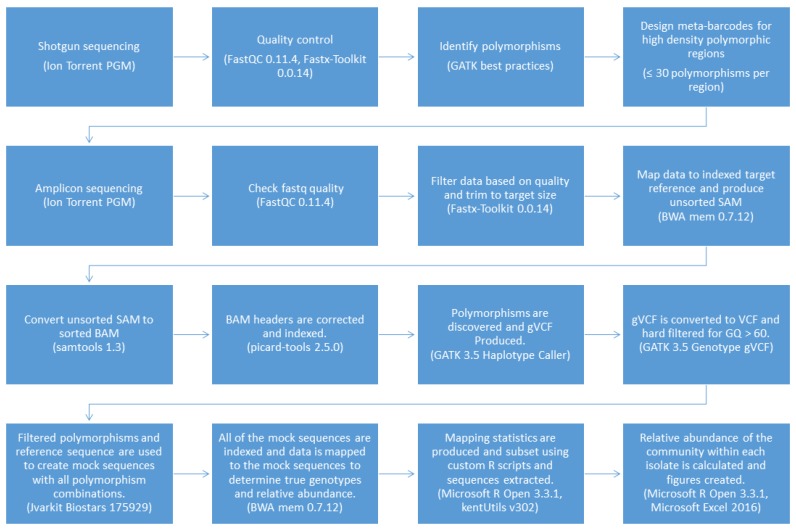
The MetaGaAP workflow to identify genotypes and the relative abundance of the community composition within a single isolate.

The pipeline was coded using Bash and Microsoft R Open 3.3.1 with a text-based interface to improve versatility and ease of use and named the Meta-barcoding Genotyping and Abundance Pipeline (MetaGaAP) [58]. A schematic of the pipeline is presented in Figure 2.

### 2.6. Comparison of amplicon and Sanger sequences

Genotype sequences identified using MetaGaAP and chromatograms from Sanger sequencing were visualized using Geneious R9.1.5 and aligned using MAFFT v7.222 (Kyoto University, Kyoto, Japan) with the FFT-NS-2 algorithm and default settings [59]. The dominant genotype and abundant minor genotypes predicted from the mapped NGS amplicon sequences were compared visually at each predicted SNP locus with the Sanger chromatographs.

## 3. Results

### 3.1. Identification of Polymorphisms in Shotgun Sequence Data

A total of 438 polymorphisms were identified within the Ion Torrent PGM shotgun dataset of the AC53 isolate, equivalent to 1 nucleotide change every 297 bases. Within the 139 ORFs in the AC53 consensus genome sequence, 37 ORFs contained no polymorphisms and 102 ORFs contained polymorphisms. Polymorphisms were identified within exons, intergenic regions and all five homologous repeat (Hr) regions (Table S1): 53 were insertions, 339 were deletions and 46 were substitutions. Most ORFs contained 9 or fewer polymorphisms. The ORF with the highest number of polymorphisms was BRO-A (30) and had a mix of substitutions, insertions and deletions and was selected for amplicon sequencing. DNA polymerase contained 5 polymorphisms across the entire 3 kb ORF: a 325 bp region within the ORF that contained no polymorphisms was selected as the negative control.

### 3.2. Validation by Comparison of Amplicon Sequence Variants to Shotgun Sequence Data

**Figure 3:**
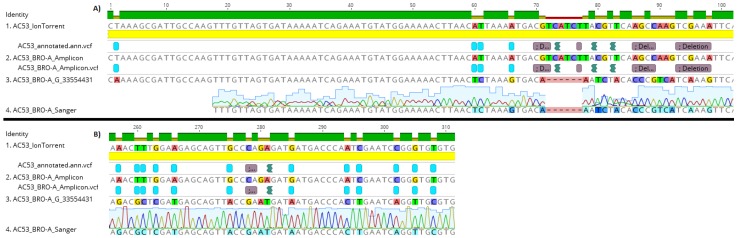
Fragments A and B of the HaSNPV-AC53 BRO-A target region containing the identified polymorphisms, showing identical alignment of polymorphisms in the amplicon, shotgun and Sanger sequences of the dominant BRO-A variant identified by MetaGaAP within the wild type baculovirus isolate HaSNPV-AC53.

AC53 shotgun sequencing predicted 25 polymorphism in the targeted ‘barcode’ region within BRO-A. All 25 polymorphisms were identified in the amplicon sequences (Figure 3). No polymorphisms were detected in the amplicon data of the DNA polymerase region, as predicted from the shotgun data (Figure S1). A single polymorphism (an ‘A’ substitution at position 293) was detected in the BRO-A amplicon sequences of the derived strain AC53-T2. This polymorphism was confirmed as one of the 25 polymorphisms in AC53 wild-type isolate shotgun and amplicon data.

### 3.3. Genotype Sequence Construction, Abundance Mapping and Validation by Sanger Sequencing

The 25 polymorphisms identified in the BRO-A amplicon ‘barcode’ region of isolate AC53 generated 3.4 × 10^7^ possible combinations of polymorphisms in reference sequences in the custom database. Mapping of the amplicon sequences data to this database identified 329 of these possible sequences were present in the amplicon sequencing, with a minimum of 1 read mapping to them. Of these, 28 amplicon sequences with between 21× and 258,084× coverage were identified (Table 2). Genotype abundance was estimated from the number of reads mapping to each of these 28 hypothetical variants.

Genotype G_33554431 accounted for 97% of the reads and was thus predicted to be the dominant genotype, while the second most abundant genotype (G_33554303) accounted for 0.62% of the reads (Table 2). The dominant genotype G_33554431 was confirmed by Sanger sequencing, with 100% sequence similarity (Figure 3).

**Figure 4:**
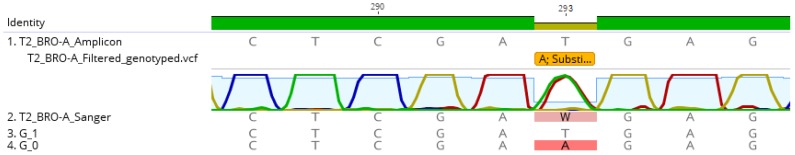
Comparison of the AC53-T2 reference sequence to the Sanger sequence and the two identified genotypes within HaSNPV-AC53-T2. The Sanger chromatogram at position 293 shows the two competing genotypes which were identified with MetaGaAP and validates relative abundance result.

The single polymorphism detected in the BRO-A amplicon sequences of the tissue-culture derived strain AC53-T2 resulted in generation of two reference sequences: with the A substitution or without the substitution (i.e., with a T). Mapping of amplicon sequence data to the two reference sequences showed that both were present in similar abundance: the T genotype accounted for 54% of reads and the A genotype for 46% of reads (Table 3). The dominant T genotype of the derived strain AC53-T2 had 100% sequence similarity with the dominant G_33554431 genotype of the AC53 wild type isolate. The minor A genotype of AC53-T2 had 100% homology to genotype G_33553919 of the AC53 wild type isolate, which accounted for only 0.05% of the reads in the wild type isolate (Table 3). The Sanger chromatogram detected both genotypes in strain AC53-T2, with both A and T detected in approximately equal intensity in the chromatogram at position 293 (Figure 4).

The single genotype predicted by the amplicon sequencing of DNA polymerase was confirmed to have 100% homology with the Sanger sequencing (Figure S1).

## 4. Discussion

Shotgun sequencing and identification of polymorphisms was used to identify of custom ‘barcode’ regions in the viral metapopulation of the wild type baculovirus isolate HaSNPV-AC53. Hr and non-coding regions were excluded to reduce possible primer bias and sequencing errors. The highest number of polymorphisms was identified in an AT-rich region of the BRO-A ORF. The high abundance of mutations in BRO-A has been previously described in published whole-genome sequences of SNPV isolates from *Helicoverpa spp.* [19,60,61] and a polymorphism rich ‘custom barcode’ region was selected within the ORF [62,63,64].

In contrast, only five polymorphisms were identified across the entire 3 kbp DNA polymerase ORF. A previous study using a different HaSNPV isolate identified 60 polymorphisms in the DNA polymerase ORF using 454 pyrosequencing with only 30× coverage, but the authors had expected DNA polymerase to be much more highly conserved [33]. Our results support this expectation and we suggest that the low coverage of the 454 pyrosequencing may have led to overestimation of polymorphisms in that study [65,66,67].

Comparison of the amplicon sequence data identified the same 25 polymorphisms in BRO-A and absence of polymorphisms in DNA polymerase, as predicted from the shotgun sequence data of HaSNPV-AC53. In contrast, a single polymorphism was predicted in the amplicon data of the tissue culture derived strain AC53-T2, which was also confirmed as one of the 25 polymorphisms predicted from the shotgun data of the parent isolate. This validates the use of amplicon and shotgun sequence to compare polymorphisms using the GATK best practices guidelines [38,39,40,68].

Comparison of amplicon data with the database of all possible combinations of polymorphisms using MetaGaAP identified 28 variants within the HaSNPV-AC53 wild type viral metapopulation at the level of 20× read coverage. A dominant variant within the wild type HaSNPV-AC53 accounted for 97% of the population. In contrast, two variants of approximately equal abundance were identified in the derived strain AC53-T2. The slightly more abundant variant in AC53-T2 had 100% sequence similarity to the dominant variant in the parent isolate, and the other variant had 100% sequence similarity to a minor variant accounting for 0.05% of abundance in the parent isolate. This supports the sensitivity of MetaGaAP to detect and identify minor variants as low as 129× coverage. We suggest including strains with a minimum 20× coverage threshold (to eliminate ‘false positives’ due to sequencing error). However, the presence of minor genotypes with coverage below 129× would require confirmation by, for example, detection in multiple deep sequencing of the isolate during different stages of infection, or large scale sequence or virus cloning and characterisation.

Sanger sequencing is the ‘gold-standard’ for validation of NGS datasets and has the lowest error rates [69]. Sanger sequencing confirmed the identification of the predicted dominant variant in both the BRO-A and DNA Polymerase amplicons of the HaSNPV wild type metapopulation. Furthermore, Sanger sequencing detected both the predicted variants within the derived strain AC53-T2 in the approximately equal proportions calculated by MetaGaAP. This confirmed the validity both of the identification of variants and the calculation of their relative abundance by MetaGaAP.

Current tools for 16S based taxonomic classification of clinical isolates use either pairwise or non-pairwise alignments to a very limited set of sequences from culture collections. Most meta-barcode analyses of microbial communities use partial regions of 16S and 18S ribosomal RNA and, to a lesser degree, the ITS region of fungi, while ‘barcodes’ for viruses are limited to a few significant virus types such as small RNA viruses [1,2,3,6,7,15,70,71]. These approaches are primarily used for taxonomic classification and rely on either phylogenetic clustering or alignment scores in comparison to sequences in reference databases such as Greengenes for 16S [9,72,73,74,75,76] However, these approaches are limited by errors such as submission of misannotated sequences or identification based on short or partial sequences, in addition to the limited sequence availability for non-model organisms [77,78,79].

## 5. Conclusions

MetaGaAP accurately identified and estimated abundance of variants in a virus metapopulation by generating a custom database from sequence data and comparison with ultra-deep sequencing of amplicons of novel, polymorphism-rich ‘barcode’ regions in the viral metagenome. However, the computer data handling and processing time increases as the number of polymorphisms increases and the number of possible combinations generated in the database increases by 2^y^, where y = number of polymorphisms. The application is thus practically limited regions with 30 or fewer polymorphisms.

Despite this limitation, MetaGaAP has potential application in analysis of community composition where suitable reference sequence databases are not available, complete or accurately assigned, and can be used to identify and quantify strain variants in pathology, ecology and evolutionary studies without the need for viral cloning. MetaGaAP is publicly available for download on GitHub [58].

## Figures and Tables

**Table 1:** Primers used for amplification of selected regions within the ORFs BRO-A and DNA polymerase.

Target Gene	Primer	Fragment Size
BRO-A	* 5′-CATTTGCAAGGATATTGGAGT-3′ ^#^ 5′-AAGCTCGTTGGTTATCACAT-3′	365 bp
DNA Polymerase	* 5′-GTATGACTTATCACGACAATTGC-3′ ^#^ 5′-CGGTTTGCATATGTACTCTG-3′	325 bp

* An adapter, BarcodeX barcode adaptor and random hexamer is attached to the forward primer in the 5′ direction; ^#^ trP1 adapter is attached to the reverse primer in the 5′ direction.

**Table 2:** Relative abundance of the identified AC53 BRO-A community composition that were above the 20x coverage threshold with G_33554431 identified as the dominant strain in the population.

Genotype	Reads	Relative Abundance %
G_33554431 ^#^	258084	97.03
G_33554303	1643	0.62
G_33552383	787	0.30
G_16777215	666	0.25
G_33554423	533	0.20
G_25165823	437	0.16
G_33554430	437	0.16
G_33292287	400	0.15
G_31457279	393	0.15
G_33554429	261	0.10
G_33554399	228	0.09
G_33554427	213	0.08
G_33553919 ^*^	138	0.05
G_33554175	129	0.05
G_33546239	123	0.05
G_33554367	105	0.04
G_29360127	103	0.04
G_33030143	103	0.04
G_33550335	92	0.03
G_33552255	68	0.03
G_33521663	62	0.02
G_33554415	56	0.02
G_33554428	55	0.02
G_20971519	52	0.02
G_33553407	48	0.02
G_23068671	35	0.01
G_33554239	28	0.01
G_33538047	21	0.01

^#^ Equivalent to the AC53-T2 BRO-A G_1; ^*^ Equivalent to the AC53-T2 BRO-A G_0.

**Table 3:** Relative abundance of the two BRO-A genotypes within AC53-T2 BRO-A.

Genotype	Reads	Relative Abundance %
AC53-T2 BRO-A G_1	104,065	54.27
AC53-T2 BRO-A G_0	87,689	45.73

## Supplementary Materials

The following are available online at www.mdpi.com/2079-7737/6/1/14/s1, Figure S1: Comparison of the AC53 DNA polymerase Sanger sequence and the AC53 DNA polymerase reference sequence showing 100% nucleotide identity and no polymorphisms identified., Table S1: Polymorphisms detected within ORFs. BRO-A has the highest number of polymorphisms (30) and HOAR and P74 have the second highest (13).

## Software and Dataset Availability

MetaGaAP is available for download at https://github.com/CNoune/IMG_pipelines. Genotypes described in this paper are available for download at https://researchdatafinder.qut.edu.au/display/n14806.
