# Supplementary material for: MetaGaAP: A Novel Pipeline to Estimate Community Composition and Abundance from Non-Model Sequence Data
Source: Biology (Basel). 2017 Feb 17;6(1):14. doi: 10.3390/biology6010014 (PMC5372007; doi:10.3390/biology6010014)
Supplement: Supplementary file 1 [file biology-06-00014-s001.pdf]

Supplementary Materials: MetaGaAP: A Novel Pipeline to Estimate Community Composition and Abundance from Non-Model Sequence Data

Christopher Noune, Caroline Hauxwell

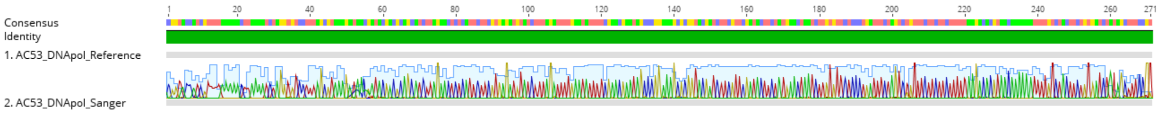

Figure S1. Comparison of the AC53 DNA polymerase Sanger sequence and the AC53 DNA polymerase reference sequence showing 100% nucleotide identity and no polymorphisms identified.

Table S1. Polymorphisms detected within ORFs. BRO-A has the highest number of polymorphisms (30) and HOAR and P74 have the second highest (13).

ORF	Polymorphisms
Exons and Intergenic Regions	45
BRO-A	30
Hr 4	21
Hr 5	19
Hr 2	16
HOAR	13
P74	13
Hr 1	12
Helicase	9
ODV-E66	9
Lef-8	8
Cathepsin	7
ORF82	7
ORF91	7
ORF105	7
Chitinase	6
ORF132	6
VP80	6
DNA polymerase	5
ORF93	5
ORF102	5
P49	5
VP39	5
EGT	4
IE-1	4
Lef-4	4
ORF64	4
ORF67	4
ORF88	4

ORF136	4
ORF137	4
ORF138	4
P26	4
Hr3	4
ALK-EXO	3
BRO-B	3
HE56	3
IAP-2	3
lef-3	3
ME53	3
ODV-EC27	3
ORF6	3
ORF13	3
ORF25	3
ORF33	3
ORF44	3
ORF76	3
ORF96	3
ORF125	3
P6.9	3
P47	3
VP91	3
FP	2
GP41	2
Hypothetical ORF	2
Lef-1	2
ODV-E56	2
ORF2	2
ORF5	2
ORF26	2
ORF27	2
ORF34	2
ORF71	2
ORF73	2
ORF75	2
ORF99	2
ORF124	2
PKIP-1	2
Polyhedrin	2
VP1054	2
38.7K protein	1
39K/PP31	1

BRO-C	1
CALYX/PEP	1
CG30	1
DBP1	1
FGF	1
GP19	1
GP37	1
IE-0	1
Lef-5	1
Lef-9	1
ODV-E25	1
ORF18	1
ORF29	1
ORF36	1
ORF37	1
ORF40	1
ORF50	1
ORF52	1
ORF61	1
ORF78	1
ORF85	1
ORF92	1
ORF97	1
ORF100	1
ORF103	1
ORF104	1
ORF106	1
ORF109	1
ORF110	1
ORF112	1
ORF118	1
ORF135	1
P10	1
PK1	1
Ubiquitin	1
VLF-1	1
ORF113	0
ORF131	0
ARIF-1	0
P24	0
Lef-2	0
ORF57	0
ORF39	0

ORF130	0
ORF30	0
Lef-6	0
ORF42	0
ORF87	0
ORF83	0
SOD	0
ORF51	0
ORF69	0
ORF128	0
ORF43	0
ORF117	0
Lef-11	0
ORF70	0
ORF101	0
ORF111	0
ORF12	0
ORF114	0
ORF45	0
Lef-10	0
ORF119	0
ORF48	0
ORF22	0
ORF49	0
ORF54	0
ODV-E18	0
ORF95	0
ORF17	0
ORF7	0

© 2017 by the authors. Submitted for possible open access publication under the terms and conditions of the Creative Commons Attribution (CC-BY) license (http://creativecommons.org/licenses/by/4.0/).
